# Intake of water and beverages of children and adolescents in 13 countries

**DOI:** 10.1007/s00394-015-0955-5

**Published:** 2015-06-14

**Authors:** I. Guelinckx, I. Iglesia, J. H. Bottin, P. De Miguel-Etayo, E. M. González-Gil, J. Salas-Salvadó, S. A. Kavouras, J. Gandy, H. Martinez, S. Bardosono, M. Abdollahi, E. Nasseri, A. Jarosz, G. Ma, E. Carmuega, I. Thiébaut, Luis A. Moreno

**Affiliations:** Hydration and Health Department, Danone Research, Palaiseau, France; GENUD (Growth, Exercise, NUtrition and Development) Research Group, Faculty of Health Sciences, Universidad de Zaragoza, Saragossa, Spain; Faculty of Health Sciences, University of Zaragoza, C/Domingo Miral s/n, Saragossa, 50009 Spain; Human Nutrition Unit, Hospital Universitari de Sant Joan de Reus, Faculty of Medicine and Health Sciences, IISPV (Institut d’Investigació Sanitària Pere Virgili), Biochemistry Biotechnology Department, Universitat Rovira i Virgili, Reus, Spain; CIBERobn (Centro de Investigación Biomédica en Red Fisiopatología de la Obesidad y Nutrición), Institute of Health Carlos III, Madrid, Spain; Department of Health Human Performance and Recreation, University of Arkansas, Fayetteville, AR USA; British Dietetic Association, Birmingham, England, UK; School of Life and Medical Services, University of Hertfordshire, Hatfield, England, UK; RAND Corporation, Santa Monica, CA USA; Hospital Infantil de Mexico Federico Gomez, Mexico City, Mexico; Department of Nutrition, Faculty of Medicine, Universitas Indonesia, Jakarta, Indonesia; Department of Nutrition Research, Faculty of Nutrition Sciences and Food Technology, National Nutrition and Food Technology Research Institute, Shahid Beheshti University of Medical Sciences, Tehran, Iran; National Food and Nutrition Institute, Warsaw, Poland; National Institute for Nutrition and Food Safety, Chinese Center for Disease Control and Prevention, Beijing, China; Department of Nutrition and Food Hygiene, School of Public Health, Peking University, Beijing, China; Centro de Estudios Sobre Nutrición Infantil, Buenos Aires, Argentina; Research Centre of Epidemiology, Biostatistics and Clinical Research, School of Public Health, Université Libre de Bruxelles, Brussels, Belgium; Club Européen des Diététiciens de l’Enfance, Brussels, Belgium

**Keywords:** Water, Beverages, Fluid intake, Children, Adolescents

## Abstract

**Purpose:**

To describe the intake of water and all other beverages in children and adolescents in 13 countries of three continents.

**Methods:**

Data of 3611 children (4–9 years) and 8109 adolescents (10–17 years) were retrieved from 13 cross-sectional surveys (47 % males). In three countries, stratified cluster sampling design was applied to randomly recruit schools classes. A quota method was applied in the other countries to randomly recruit participants. Details on the intake of all fluid types were obtained with a fluid-specific record over 7 consecutive days.

**Results:**

In the total sample, the highest mean intakes were observed for water (738 ± 567 mL/day), followed by milk (212 ± 209 mL/day), regular soft beverages (RSB) (168 ± 290 mL/day) and juices (128 ± 228 mL/day). Patterns characterized by a high contribution of water, RSB or hot beverages to total fluid intake were identified among the countries with close geographical location. Adolescents had a significantly lower milk intake and higher intake of RSB and hot beverages than children in most countries. The most consistent gender difference observed was that in both age groups males reported a significantly higher RSB consumption than females.

**Conclusion:**

On average, water was the fluid consumed in the largest volume by children and adolescents, but the intake of the different fluid types varied substantially between countries. Since the RSB intake was as large, or even larger, than water intake in some countries, undertaking actions to improve fluid intake habits of children and adolescents are warranted.

**Electronic supplementary material:**

The online version of this article (doi:10.1007/s00394-015-0955-5) contains supplementary material, which is available to authorized users.

## Introduction

The World Health Organization (WHO) has raised concern regarding an excessive intake of sugar-sweetened beverages (SSB) for children [1]. This concern is based on a meta-analysis of long-term prospective cohort studies concluding that children consuming the largest intakes of SSB had greater likelihood of being overweight or obese than children with the lowest intakes [2]. Moreover, the sugars present in SSB have been associated with dental carries prevalence both in children and adults [2, 3]. Consequently, the WHO has set recommendations for intake of free sugars to <10 % of total energy intake and suggests a further reduction to <5 % of total energy intake [1]. Evidence has suggested that a reduction in energy intake facilitating weight management can be achieved among regular SSB consumers if they replace their SSB with drinking water [4, 5].

Now that this recommendation has been made, surveying populations is globally needed to assess the intake patterns of different fluid types (water and all other beverages). Several large cohort or cross-sectional studies have already reported the intake of different fluid types in children [6–9]. In 2014, Özen et al. [10] published a systematic review of studies assessing beverage consumption across age groups. In children, plain water contributed up to 58 % of total beverage intake, with great variability from 21 to 58 % between countries [10]. This number was increased to 51–75 % in adolescents. Surprisingly, even though the studies included in this review assessed beverage consumption, they did not all report the intake of water [10]. Furthermore, other inconsistencies were noted in the study design, dietary assessment methods, classification of beverages and age categories which limit the comparison of results between countries. To make an inter-country comparison, a review containing surveys which assessed fluid intake with the same methodology and reported intake of all fluids would be needed. Ideally, the sample of such surveys would be representative of the national sample and would cover the same and wide age range. The review by Özen et al. [10] showed also that for one country, such as the USA, several studies reporting on fluid intake of children are available. However, fluid intake of children and adolescents remains to be assessed in numerous countries worldwide. The aim of the present pooled analysis was therefore to describe the intake pattern of water and all other beverages in children and adolescents, aged 4 up to 17 years in 13 countries of three continents. Differences in intakes between sex and age groups are also reported.

## Methods

### Design and study population

This pooled reanalysis was performed on the individual data of participants aged 4–17.9 years of 13 cross-sectional surveys. The primary objective of all surveys was to assess the intake of drinking water and different types of beverages. The secondary objective was to assess the barriers or believes individuals have about the consumption of water or other fluid types. The surveys included in the pooled reanalysis were conducted in Latin America (Mexico, Brazil, Argentina, Uruguay), Europe (Spain, France, Belgium, UK, Poland, Turkey) and Asia (Iran, China, Indonesia) between 2008 and 2014, either by private research organizations, or by the Université libre de Bruxelles/the Club Européen des Diététiciens de l’Enfance (CEDE), by the Iranian National Nutrition and Food Technology Research Institute (NNFTRI) or by the Chinese Center for Disease Control (CDC). All surveys were initiated by or in collaboration with Danone Research. The individual surveys called Liq.In^7^ (abbreviation of *Liq*uid *In*take over 7 days) took place between 2008 and 2014.

This pooled reanalysis contained both original and published fluid intake data. The protocol of the published surveys has been described in detail elsewhere [11–15]. Annex 1 summarizes the sampling method, the exclusion criteria, the period of data collection, the age range of recruited participants and the dietary assessment method of the retrieved cross-sectional surveys performed among children, adolescents and adults. Data of adults are reported elsewhere [16, 17]. Data collection was organized during a period of the year with an expected mild climate (spring or fall) in order to minimize the effect of temperature as much as possible. In brief, the surveys performed in Belgium, Iran and China used a comparable recruitment method: entire school classes were recruited via a random, stratified cluster sampling. The school classes were stratified for school grade or age of participants, regions of the country and the type of educational system. The survey in Belgium therefore focussed on the age range of 8–13 years and in Iran and China 8–17 years. Parents of the recruited school children received information on the study via parent meetings, written information sheet or phone calls. Surveys conducted in the 10 other countries randomly recruited participants with a quota-based method. Quotas were set for age, gender, region of the country, habitat and/or socioeconomic characteristics. Parents were contacted via a database of individuals volunteering to population surveys or via a systematic door-to-door approach with an invitation for their child to participate.

All parents and children willing to participate in the survey received detailed information about the survey objectives, what was expected from them, as well as a disclosure of the survey’s provisions to preserve confidentiality, risks and benefits, and a clear explanation about their option to participate voluntarily or not in the survey. After offering a detailed description of the survey, parents were asked for their oral approval to let their child participate. No monetary incentive was offered for taking part in the survey. All data were recorded in an anonymous way. Therefore, participants cannot be identified, directly or through identifiers linked to the participants. The survey protocol of the unpublished surveys was reviewed and approved by the University of Arkansas Review Board (ref. 14-12-376).

### Assessment of fluid intake

A fluid-specific record was provided to participants of all surveys in order to collect information on all their fluid consumption over 7 consecutive days. These 7-day fluid records and the associated written information were presented to the participants in the official language of the country in a paper format, except for participants in France who filled in their fluid record online. An investigator delivered and explained the fluid record to the participants during a face-to-face interview at home. For children younger than 12 years, the primary care giver of the child was requested to complete the fluid record. After 7 days, a second home visit of the investigator took place to collect the fluid record and to ensure a complete record. Surveys performed in Belgium, Iran and China deviated from this protocol as they recruited school classes [13, 14]. In these cases, both parents and teachers were involved in the completion of the fluid record. All questionnaires were verified by the researchers upon completion, and incomplete answers were clarified at the next visit.

The 7-day fluid records in all surveys were structured in order to capture the same type of information on the fluids consumed. Besides an introduction with instruction on the completion of the record, the 7-day fluid record consisted of blank tables, one for each day. Participants were instructed to complete a line in the table every time they drank anything, at any time of the day both inside their home and outside. To remind them of consumptions throughout the day, the following moments were indicated in the table: before breakfast, during breakfast, at mid-morning (between breakfast and lunch), during lunch, between lunch and afternoon tea, during afternoon tea, in the afternoon (between afternoon tea and dinner), during dinner, after dinner/before going to sleep and late at night/at dawn. For each consumption, the following questions had to be registered in the table:

- *Fluid type: What did you drink in or outside of your home?* A list of fluids types that corresponded to the one presented in annex 2 was provided.
- *Brand: What brand/flavour/packaging type did you consume?* A list of fluids and brands was provided.
- *Container: From which container did you drink?* A photographic booklet of standard containers of fluids was provided, and in China also an additional scaled water container.
- *Quantity: How much did you drink?* A code that corresponded to the number of whole units and/or fractions of the container had to be registered.
- *Form of Consumption: How did you consume the fluid?* E.g. Alone or did you mix with other products (e.g. concentrated juice syrup, energy drinks)? With which ones? Was the fluid hot or cold (with or without ice cubes)? Participants could also indicate whether or not they added sugar to their fluid.
- *Company: With whom did you consume the drink?* A list of options was presented
- *Activity: What were you doing while you were drinking?* A list of possible activities was provided.
- *Location: Where did you drink?* A list of possible locations was provided.
- *Reasons for consumption: For what reasons did you drink that fluid at that time?* From a list of options participants could record maximum three reasons.

### Classification of fluid types

The fluids recorded in all surveys were classified into: water (tap and bottled water), milk and milk derivatives, hot beverages (coffee, tea and other hot beverages), juices, regular soft beverages (RSB) (sugared and artificially sweetened, carbonated and non-carbonated soft drinks, energy drinks, sports drinks, other sugared or artificially sweetened soft drinks), alcoholic drinks and other beverages. A more detailed classification can be found in annex 2 of this paper. In five out of the 13 countries, the intake of artificially sweetened/diet beverages was separated from RSB. Since the mean intake of this fluid type was on average 7 mL/day, these fluids were included in the class of RSB to create homogeneity in the classification. In Argentina, Iran and Indonesia, only non-alcoholic beverages were recorded. In Belgium, coffee and tea intake was not recorded. However, soup intake was recorded and was classified into other beverages. In Spain and France, no fluids were classified into the group “other beverages”. Additions (e.g. sugar or honey) by hand by the participant to a fluid were not taken into account while classifying a fluid. Total fluid intake (TFI) was defined as the sum of all categories previously described. For the each category, the age-, sex- and country-specific means of the absolute intakes over the 7 days were calculated.

### Anthropometric data

Height in metres (m) and weight in kilograms (kg) were measured by the investigator in the surveys of Belgium, Poland, Iran and China and self-reported in the other surveys. No anthropometric data were collected in Mexico, Brazil, Uruguay, Argentina and Indonesia. When weight and height measures were available, body mass index (BMI) was calculated (in kg/m^2^) and reported with the intention solely to describe the study samples. The proportion of male and female participants with underweight, normal weight, overweight and obesity as well as the socio-economic status of the participants has been described elsewhere [11–15].

### Statistical analysis

The same data cleaning was applied to the individual data of all 13 surveys. Participants who did not complete the full 7-day fluid intake record or who reported the exactly same intakes on ≥2 days over the 7 day period were excluded from the analysis, as well participants reporting a mean total daily fluid intake below 0.4 L/day or higher than 4 L/day as they are considered to be non-plausible intakes. The final sample size for this analysis was 11,720 participants, who were classified into children (4–9.9 years) and adolescents (10–17.9 years).

Continuous and categorical data are presented as mean (SD) and percentage (*n*), respectively. In annex 3 of this paper, standard error of the mean (SEM), median and additional percentiles (5th, 10th, 25th, 75th, 90th, 95th) of the 7-day intakes of fluids are also reported. The effect of age and sex on the intake of the different beverages types was tested with a student’s *t* test. Analyses were performed using the JMP software version 10.0.0 (SAS Institute Inc., Cary, NC). All statistical tests were two-tailed, and the significance level was set at *p* < 0.01 to correct for the multiple testing.

## Results

Table 1 describes the sample size, age and BMI of both age groups by country. The proportion of children and adolescents in the sample of each country was balanced, except for Belgium, Iran and China. In the latter three samples, 68–80 % of the participants were adolescents. Consequently, the mean age of the children in these three samples was higher than the mean age of the children in the other samples.

**Table 1:** General characteristics of the children and adolescent samples, categorized by country and gender

Country	Sex	Children			Adolescents		
		Sample size	Age	BMI	Sample size	Age	BMI
		*n* (%)	Mean (SD)	Mean (SD)	*n* (%)	Mean (SD)	Mean (SD)
Mexico	Male	234 (60)	6.52 (1.7)	ND	172 (56)	12.35 (2.3)	ND
	Female	153 (40)	6.58 (1.8)	ND	134 (44)	12.62 (2.3)	ND
Brazil	Male	183 (52)	6.30 (1.6)	ND	212 (49)	13.75 (2.3)	ND
	Female	166 (48)	6.18 (1.6)	ND	218 (51)	13.51 (2.4)	ND
Uruguay	Male	30 (50)	6.50 (1.7)	ND	38 (48)	12.87 (1.9)	ND
	Female	30 (50)	6.70 (1.5)	ND	41 (52)	13.93 (2.1)	ND
Argentina	Male	30 (34)	6.27 (1.6)	ND	44 (42)	14.57(2.0)	ND
	Female	59 (66)	6.47 (1.7)	ND	60 (58)	13.57 (2.6)	ND
Spain	Male	43 (51)	6.33 (1.5)	16.60 (2.8)	63 (54)	13.38 (2.3)	21.30 (3.4)
	Female	42 (49)	6.12 (1.7)	17.28 (2.9)	53 (46)	13.19 (2.1)	20.82 (3.7)
France	Male	119 (58)	6.34 (1.7)	15.74 (2.4)	92 (48)	12.24 (1.6)	19.92 (3.2)
	Female	87 (42)	6.48 (1.8)	16.07 (2.9)	101 (52)	12.27 (1.6)	18.54 (3.6)
Belgium	Male	116 (44)	8.82 (0.4)	17.37 (2.5)	259 (45)	11.02 (0.9)	18.91 (3.6)
	Female	150 (56)	8.88 (0.3)	17.14 (2.7)	315 (55)	10.96 (0.9)	18.90 (3.5)
UK	Male	67 (45)	6.61 (1.6)	21.58 (3.7)	90 (43)	12.51 (2.0)	21.58 (6.3)
	Female	81 (55)	6.70 (1.5)	20.80 (8.6)	120 (57)	12.89 (82.0)	19.69 (3.9)
Poland	Male	80 (52)	6.33 (1.6)	16.72 (3.1)	90 (51)	13.36 (2.5)	19.81 (3.7)
	Female	74 (48)	6.70 (1.5)	16.70 (3.4)	86 (49)	12.47 (2.1)	18.70 (3.5)
Turkey	Male	16 (10)	6.56 (2.0)	19.26 (4.0)	51 (24)	14.96 (2.0)	21.38 (3.7)
	Female	148 (90)	6.42 (1.7)	18.57 (5.23)	161 (76)	12.61 (2.1)	18.85 (3.5)
Iran	Male	84 (47)	8.67 (0.7)	18.41 (4.7)	283 (47)	13.95 (2.1)	21.49 (4.7)
	Female	93 (53)	8.75 (0.7)	18.40 (4.5)	324 (53)	14.22 (2.1)	21.83 (4.3)
China	Male	540 (48)	8.50 (0.5)	17.17 (2.8)	2165 (48)	13.03 (2.1)	19.61 (3.7)
	Female	580 (52)	8.55 (0.5)	16.35 (2.6)	2342 (52)	13.20 (2.1)	18.82 (3.1)
Indonesia	Male	200 (49)	6.57 (1.7)	ND	243 (41)	13.16 (2.3)	ND
	Female	206 (51)	6.39 (1.7)	ND	352 (59)	13.60 (2.2)	ND
Total^a^	Male	1742 (48)	7.35 (1.7)	17.21 (3.3)	3802 (47)	13.00 (2.2)	19.85 (3.9)
	Female	1869 (52)	7.41 (1.7)	17.09 (3.9)	4307 (53)	13.01 (2.2)	19.19 (3.5)

*BMI* body mass index, *ND* no data

^a^Includes only data of countries with available data on the presented characteristics

In general, across the total samples of the countries, the highest daily intakes were observed for water (738 ± 567 mL/day), followed by milk (212 ± 209 mL/day), RSB (168 ± 290 mL/day) and juices (128 ± 228 mL/day). There was a large inter-country variation in the intake of a given fluid type. Daily water intake ranged from 296 mL/day in Poland to 1516 mL/day in Indonesia, whereas daily milk intake ranged from 123 mL/day in Indonesia to 530 mL/day in Uruguay. The intake of RSB and juices ranged from 64 mL/day in China to 625 mL/day in Argentina and from 21 mL/day in Indonesia to 555 mL/day in Brazil, respectively. The age-, sex- and country-specific means of intake of the different fluid types are presented in Tables 2 and 3.

**Table 2:** Mean daily intake of different fluid types (mL/day) of children (4–9 years), stratified by country

Country	Sex	Water	Milk	Hot beverages	Juices	RSB	Alcoholic beverages	Other beverages
Mexico	Male	424 (409)	350 (239)	23 (76)	155 (207)	424 (416)	0 (0)	15 (164)
	Female	410 (409)	321 (207)	35 (75)	157 (216)	357 (316)	0 (0)	4 (22)
Brazil	Male	536 (397)	487 (259)^a^	33 (99)	479 (335)	136 (216)	0 (0)	4 (27)
	Female	543 (317)	428 (284)	45 (106)	497 (369)	141 (239)	0 (0)	8 (42)
Uruguay	Male	751 (539)	541 (216)	11 (37)	380 (428)	406 (387)	0 (0)	40 (185)
	Female	896 (474)	603 (213)	15 (47)	388 (506)	447 (465)	0 (0)	0 (0)
Argentina	Male	312 (339)	341 (149)	99 (140)	303 (397)	582 (472)	ND	0 (0)
	Female	420 (633)	353 (180)	157 (361)	310 (368)	616 (709)	ND	2 (9)
Spain	Male	804 (549)	574 (279)^a^	37 (120)	250 (399)	119 (174)	0 (0)	ND
	Female	785 (501)	440 (264)	36 (86)	174 (159)	66 (106)	0 (0)	ND
France	Male	546 (295)	245 (159)	3 (16)	86 (94)	145 (195)	0 (0)	ND
	Female	529 (325)	245 (184)	4 (13)	95 (104)	138 (173)	0 (0)	ND
Belgium	Male	379 (293)	167 (160)	ND	139 (120)	195 (210)^a^	0 (0)	16 (33)
	Female	371 (624)	135 (149)	ND	126 (142)	138 (158)	0 (3)	25 (42)
UK	Male	529 (348)	241 (253)	31 (95)	238 (220)	652 (605)^b^	0 (0)	1 (7)
	Female	434 (297)	281 (306)	67 (142)	261 (297)	407 (363)	0 (0)	0 (4)
Poland	Male	252 (297)	164 (139)	460 (241)	191 (187)	324 (250)	0 (4)	26 (117)
	Female	267 (347)	143 (138)	472 (274)	213 (171)	263 (252)	0 (4)	7 (18)
Turkey	Male	773 (297)	268 (232)	222 (154)	86 (103)	217 (341)	0 (0)	240 (247)
	Female	851 (443)	275 (315)	207 (181)	129 (143)	113 (194)	0 (0)	159 (244)
Iran	Male	709 (347)^a^	271 (173)	137 (99)^b^	80 (86)	117 (95)	ND	2 (7)
	Female	610 (292)	288 (167)	99 (88)	70 (73)	91 (82)	ND	6 (21)
China	Male	660 (380)	185 (164)	11 (57)^a^	47 (101)	65 (114)^c^	ND	15 (40)
	Female	641 (374)	187 (157)	19 (71)	41 (82)	44 (84)	ND	19 (45)
Indonesia	Male	1387 (746)	232 (276)^b^	90 (179)	21 (70)	191 (375)	ND	18 (59)
	Female	1394 (740)	163 (249)	72 (120)	22 (73)	164 (300)	ND	10 (36)
Total	Male	651 (535)	272 (238)^b^	54 (143)^b^	144 (235)	204 (323)^c^	0 (1)	14 (83)^b^
	Female	661 (525)	247 (236)	70 (159)	140 (235)	161 (280)	0 (2)	23 (87)

Intake data presented as mean (SD) and analysed with a Student’s *t* test

*ND* no data, *RSB* regular soft beverages

^a^ *p* value <0.05; ^b^ *p* values <0.01; ^c^ *p* values <0.0001

**Table 3:** Mean daily intake of different fluid types (mL/day) of adolescents (10–17 years) stratified by country

Country	Sex	Water	Milk	Hot beverages	Juices	RSB	Alcoholic beverages	Other beverages
Mexico	Male	432 (430)	337 (280)	53 (116)	172 (230)	461 (413)	2 (16)	4 (23)
	Female	531 (622)	330 (288)	63 (112)	165 (197)	479 (512)	1 (11)	3 (15)
Brazil	Male	720 (587)	312 (261)	107 (199)	589 (488)	293 (390)	8 (120)	1 (14)
	Female	653 (488)	345 (275)	105 (196)	629 (438)	242 (326)	12 (92)	2 (18)
Uruguay	Male	863 (583)	551 (293)	29 (93)^a^	300 (337)	684 (492)	31 (107)	0 (0)
	Female	773 (570)	451 (249)	251 (544)	437 (420)	638 (536)	41 (169)	16 (66)
Argentina	Male	249 (296)	232 (187)	247 (304)	366 (531)	732 (976)	ND	7 (24)
	Female	309 (346)	219 (169)	393 (543)	219 (267)	578 (558)	ND	7 (38)
Spain	Male	898 (568)	365 (229)	43 (113)	173 (219)	314 (443)	9 (49)	ND
	Female	913 (672)	377 (281)	49 (113)	220 (253)	191 (214)	2 (14)	ND
France	Male	642 (320)	269 (195)^a^	42 (130)	111 (90)	282 (274)^a^	2 (8)	ND
	Female	617 (333)	203 (172)	38 (104)	110 (108)	199 (212)	0 (2)	ND
Belgium	Male	419 (373)	154 (138)^a^	ND	150 (175)	245 (229)^b^	1 (6)	19 (46)
	Female	422 (341)	129 (125)	ND	141 (151)	195 (195)	0 (1)	26 (57)
UK	Male	457 (426)	195 (206)	141 (257)	263 (355)	693 (574)^a^	14 (92)	7 (38)
	Female	506 (466)	157 (205)	151 (263)	246 (298)	525 (436)	7 (39)	4 (18)
Poland	Male	302 (305)	109 (116)	465 (327)	188 (201)	346 (324)	9 (40)	26 (133)
	Female	356 (327)	129 (158)	514 (265)	191 (182)	303 (290)	2 (17)	14 (53)
Turkey	Male	999 (643)	145 (221)	253 (171)	119 (166)	209 (274)	2 (15)	255 (283)
	Female	927 (492)	207 (247)	260 (233)	131 (204)	161 (181)	0 (0)	195 (245)
Iran	Male	709 (432)^a^	241 (184)	222 (149)	57 (76)	185 (165)^c^	ND	8 (24)
	Female	631 (363)	218 (187)	199 (157)	54 (69)	116 (111)	ND	9 (31)
China	Male	869 (532)^c^	167 (163)^c^	23 (94)^c^	73 (137)	88 (147)^c^	ND	17 (55)^c^
	Female	715 (440)	185 (152)	34 (114)	70 (117)	48 (88)	ND	25 (58)
Indonesia	Male	1621 (872)	73 (140)	129 (224)	13 (41)^a^	200 (334)	ND	13 (53)
	Female	1589 (812)	74 (166)	101 (168)	27 (87)	203 (389)	ND	17 (77)
Total	Male	813 (600)^c^	191 (195)	69 (165)^c^	123 (234)	185 (305)^c^	6 (65)	17 (67)^c^
	Female	740 (556)	191 (189)	83 (189)	120 (216)	141 (264)	4 (51)	26 (79)

Intake data presented as mean (SD) and analysed with a Student’s *t* test

*ND* no data, *RSB* regular soft beverages

^a^ *p* value <0.05; ^b^ *p* values <0.01; ^c^ *p* values <0.0001

**Fig. 1:**
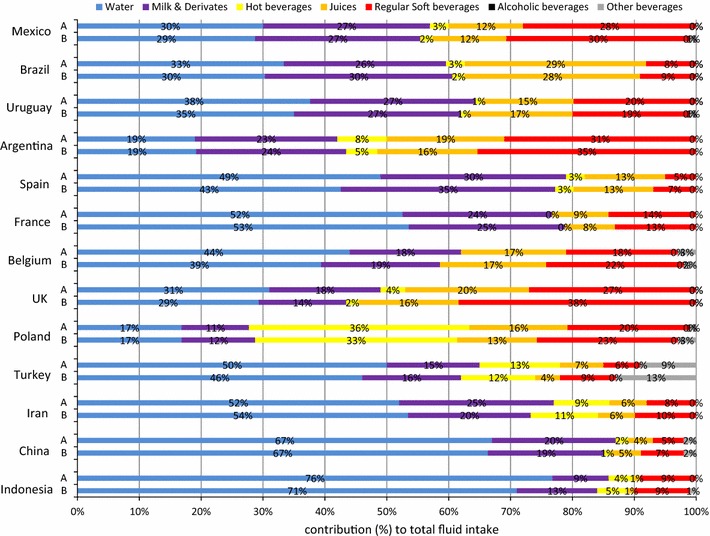
Contribution (%) of the different fluid types to total fluid intake of children (4–9.9 years) stratified by country and gender with being *A* female and *B* male

Despite these large differences in volumes of intake of the different fluid types, some samples had comparable patterns of contribution of fluid types to TFI in the children (Fig. 1). The intakes of the Chinese and Indonesian sample were characterized by the largest contribution of water to TFI (respectively, 67 and 73 % in total children sample) of all samples. In the samples of Spain, France, Belgium, Iran and Turkey, half of the TFI came from water (42–53 % in total children sample). In these five samples, the other fluids contributed for a similar amount to TFI, except for the hot beverages. In the total children sample of Iran and Turkey, hot beverages contributed, respectively, for 10 and 13 % to TFI, whereas in the French and Spanish sample they contributed only for 0–3 %. Large contributions of hot beverages to TFI were also reported in the total children sample of Poland (34 %) and Argentina (13 %). Besides the large contribution of hot beverages, these two samples were also characterized by a contribution of RSB to TFI (22–35 %) that was larger than the contribution of water to TFI (19–21 %). Similar results were observed in the total children sample of Brazil, Mexico and UK: in Brazil the contribution of juices to TFI (29 %) and in Mexico and UK the contribution of RSB to TFI (respectively 29–32 %) were as large as the contribution of water to TFI (29–36 %).

**Fig. 2:**
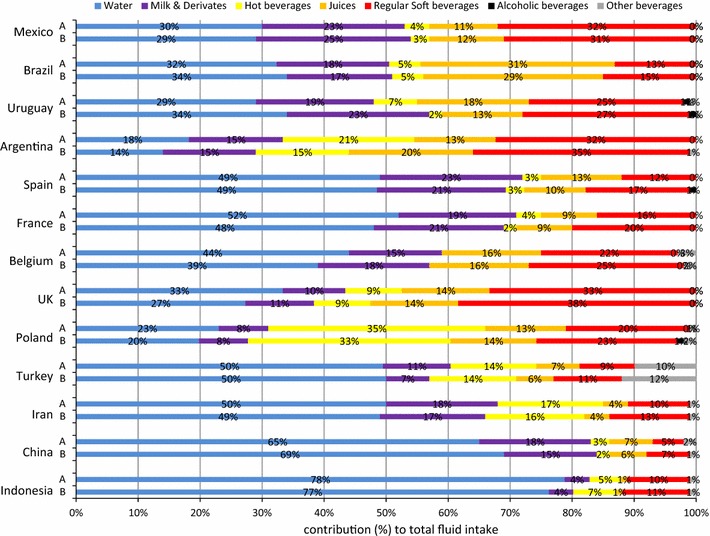
Contribution (%) of the different fluid types to total fluid intake of adolescents (10–17.9 years) stratified by country and gender with being *A* female and *B* male

Similar patterns in the contribution of the different fluid types to TFI were identified among adolescents (Fig. 2). However, a comparison between the intake of children and adolescents indicated significant age effects (all with *p* value <0.001). The most consistently observed age effect was regarding the contribution of milk to TFI: adolescents in all samples except in Belgium and Mexico had a significantly lower milk intake than children. Moreover, adolescents had a significantly higher contribution of RSB to TFI than children in Brazil, Uruguay, Spain, Turkey and Iran. In the sample of Iran, children had a higher contribution of juices to TFI than adolescents, whereas in the Chinese sample the opposite was observed. The contribution of hot beverages to TFI was significantly higher among adolescent than among children in the sample of Brazil, Uruguay, Argentina, France Iran and China. The contribution of water to TFI was comparable between children and adolescents, except in the sample of Indonesia.

Significant gender differences in the contribution of the fluid types to TFI were observed in the individual samples, yet they were inconsistent. The contribution of water to TFI was significantly higher for females in the Belgian sample (*p* = 0.0057), whereas it was lower in the Chinese samples compared with males (*p* = 0.0001). The milk contribution to TFI was higher for females in the Chinese samples, but lower in the Indonesian sample (*p* = 0.004) than for males. The contribution of other beverages to TFI was significantly higher for females in the Chinese and Belgian sample than for males (*p* < 0.0001 and *p* = 0.0026, respectively). In the Chinese sample, females also had a significantly higher contribution of hot beverages compared with males (*p* < 0.0001). The only gender difference that was consistent across several samples was observed for the contribution of RSB to TFI: males had a significantly higher RSB contribution than females in the samples of Belgium, UK, Iran and China (*p* < 0.01 for all).

When analysing the gender difference within the two age categories, significant gender differences were also observed. Among children (Fig. 1), the contribution of milk to TFI was significantly higher among men than among women in the Brazilian sample (*p* = 0.01); however, in the Iranian sample, the effect was the opposite direction (*p* = 0.005). The Chinese females drank more hot beverages than males (*p* = 0.01). Male children in UK and China had a significantly higher RSB contribution to TFI than females (*p* = 0.01 and *p* = 0.0001, respectively). In the Belgian samples, the females had a higher contribution of other beverages than males (*p* = 0.01). Among adolescents (Fig. 2), most gender differences were observed in the Chinese sample: males had a significantly higher contribution of water and RSB to TFI than females (*p* < 0.0001 for both), whereas females had a significantly higher contribution of milk, hot beverages and other beverages (all *p* < 0.0001) to TFI than males. In the Iranian sample, adolescent males had a higher contribution of RSB to TFI than adolescent females (*p* = 0.0002).

## Discussion

This unique pooled analysis of individual data of 13 cross-sectional surveys provides novel insights on fluid intake for countries that, to the best of our knowledge, had no internationally published data so far. Since all 13 surveys used the same method for data collection (a 7-day fluid-specific record), this pooled analysis gave the opportunity to observe differences in intake patterns between samples of different countries. While the range in mean milk and RSB intake between samples with the lowest and largest intake volumes was 407 and 561 mL/day, respectively, the range in mean water intake reached 1220 mL/day. These large differences in mean intake are not unusual as shown in a review from Özen et al. [10] reporting an inter-country range in TFI of 1.2 L/day (0.6–1.8 L/day), with the water contribution ranging from 21 to 58 %. Moreover, the intakes estimated for the total adolescent sample in this study are very much in line with those reported by Duffey et al. [18] in adolescents of eight European countries. It is worth noting that the intake of water was higher and the intake of RSB and alcoholic beverages lower in the current analysis compared with that of Duffey et al. [18]. Discrepancies in methodologies of recording and classification of the sugar-sweetened beverages may explain this difference.

The large differences in intakes of the different fluid types between the samples may be partly explained by differences in climate. Indeed, temperature, humidity and seasonality influence both volume consumed and the preference for certain fluid types [19, 20]. Since the surveys reported here were not designed to explore inter-country variability, no data on temperature or humidity were gathered during the period of data collection nor were seasonality taken into account. Other possible explanations for the large inter-country differences observed are cultural habits and geographical location. A certain fluid type might be consumed more out of tradition in a country (e.g. tea in UK), but also certain foods or the amount of a nutrient consumed in a certain country may influence fluid intake [21]. Samples of countries with a similar geographical location indeed showed similarities in the contribution of the different fluid types to TFI. All samples of Latin America, Mexico, Brazil, Uruguay and Argentina were characterized by a high contribution of RSB and juices to TFI. Argentina, however, differed from the other three Latin American countries by a larger contribution of hot beverages to TFI, more specifically the traditional Mate. The samples of the Asian countries included in this analysis (Indonesia and China) and also those from countries relatively closely located around the Mediterranean Sea (France, Spain, Iran and Turkey) had a comparable pattern: at least half of the fluid intake in these countries came from water. The contributions of the fluid types to TFI observed in the Belgian sample seemed comparable to the pattern observed in the samples of the Latin American countries. However, hot beverages were not recorded in the Belgian survey, and therefore, a comparison with other countries should not be made. Data in adult samples also showed similar contributions of the different fluids types to TFI in countries from the same geographical area [16]. This is not surprising since a number of studies have also shown that parental food preferences and nutrient intake including SSB are adopted by children and adolescents [22–24]. This observation suggests that there is a risk of relaying detrimental food and beverage intake habits between generations. This remains to be confirmed for fluid intake in the future.

Differences in the contribution of fluid types to TFI were observed between the two age groups, which have been reported by others [10, 18]. Among children, the intake of milk and juice was higher than among adolescents, whereas adolescents consumed more water, hot beverages, RSB and alcoholic beverages. In the total sample, both the volume and the contribution to TFI of RSB were significantly higher among children than among adolescents; however, when each sample was considered individually, adolescents always had a higher RSB intake than children. However, due to the significant differences in intake patterns between the samples and due to the unbalance in sample sizes of the countries, interpretation of the pooled data of the total sample should be done with caution. Nevertheless, among European adolescents aged 12.5–17.5 years, similar age effects on fluid intake were observed [18]. Özen et al. [10] also drew similar conclusions in their systematic review: milk intake was higher among children and was replaced by regular fluid/soft drinks among adolescents. They also reported that with age the intake of hot beverages and diet beverages increased.

The effect of gender on the intake of the different fluids was neither consistent nor very pronounced in the samples, except for the contribution of RSB to TFI. In four samples, males consumed more RSB than females. This observation suggests that females start adopting healthier hydration habits than males during adolescence, potentially due to an increased health consciousness or attention to their body image [25]. This lack of consistent gender effect on the intake of the different fluids was not in line with what has been reported previously. In a large European adolescent sample, males clearly had a higher contribution of high fat milk, SSB and alcoholic beverages and lower contribution of water than females [18]. The review by Özen et al. [10] reported also that males had a higher milk consumption than females. Until the gender effect on the intake of the different fluids has been analysed again by future surveys, it is recommended to interpret the gender and age effects country-by-country.

In eight out of the 13 samples included in this analysis, the combined mean of juices and RSB of both consumers and non-consumers was higher than 335 g/day. Intervention studies and cohort studies have shown that children and adolescents consuming SSB on a daily basis are at increased risk of becoming overweight or obese compared with non-regular consumers [26, 27]. Adolescent females who consumed more than 335 g/day had a greater overall cardio-metabolic risk, independent of their weight status (OR 3.2; 95 % CI 1.6, 6.2) (all *p*-trend ≤0.001) [27]. In light of the current prevalence of obesity and diabetes, health promotion strategies should focus, among others, on reducing the intake of SSB and increasing the intake of water. The large differences in intake patterns across samples indicate that in some countries the concern about an excessive SSB intake is higher than in other countries. Though only in three samples water was the majority of fluid intake, the concern is global.

All fluid intake data used in this analysis were self-reported. For children younger than 12 years, the primary care giver was responsible for filling the 7-day fluid record. Therefore, the risk of over- or underestimation of intake and a possible reporting error by the primary care giver cannot be excluded. In future studies, combining the recording of the intake with the collection of urine biomarkers may give an indication of the accuracy of the intake reporting. Also a validation of accuracy and reliability of the 7-day fluid record would be useful. It would also allow an estimation of the hydration status of children and adolescents. An estimate on how fluid intake contributed to the whole diet also cannot be made due to lack of food data. However, evidence suggested that a fluid-specific record might more accurately estimate fluid intake compared with a food and fluid record [28]. Since the primary aim of all 13 surveys was to assess fluid intake, the preference was given to record fluids only. Another limitation to acknowledge is that not all samples were necessarily representative of the national target sample of the country. Nevertheless, the methods of recruitment used in the surveys are recognized as valuable methods to provide enough sample by age of participants, regions of the country and different socio-economic groups for meaningful analysis. In the future, it would also be recommended to avoid the minor differences in the fluid classification that were currently present across countries.

This pooled reanalysis of individual data has several strengths. All surveys used a fluid-specific record over 7 consecutive days and are therefore assumed to provide data highly representative of habitual daily intakes. Moreover, all records were supported by a photographic booklet to increase accuracy of the reported volumes. An additional strength is that this compilation of 13 samples of different countries allowed to highlight the large diversity in fluid intake patterns across countries.

In conclusion, this analysis answers sorely to the need of data on fluid intake patterns for children and adolescents from various countries. The data indicated variability in intake patterns by age and sex. Additionally, they indicated a prevalent consumption of caloric fluids including juices and RSB. Water accounted for less than half of TFI for a large proportion of the children and adolescents. Considering that water is the preferred fluid, the data warrant further work to understand the variability across countries and to efficiently increase water intake of children and adolescents. Creating a *hydrogenic* environment for the child or adolescent could be one action, among others, to increase the adherence to the WHO recommendation on energy intake of free sugars.

## Electronic supplementary material

Supplementary material 1 (DOCX 23 kb)

Supplementary material 2 (DOCX 17 kb)

Supplementary material 3 (DOCX 49 kb)
