# Supplementary material for: Intake of water and beverages of children and adolescents in 13 countries
Source: Eur J Nutr. 2015 Jun 14;54(Suppl 2):69–79. doi: 10.1007/s00394-015-0955-5 (PMC4473084; doi:10.1007/s00394-015-0955-5)
Supplement: Supplementary file 1 — Supplementary material 1 (DOCX 23 kb) [file 394_2015_955_MOESM1_ESM.docx]

**Annex 1.** General characteristics (a) and data collection (b) of 13 cross-sectional surveys aiming to record intake of specific fluids (water and beverages of all kind) of children, adolescents and/or adults

| 1. General characteristics of cross-sectional surveys | | | | | | | | | |
| --- | --- | --- | --- | --- | --- | --- | --- | --- | --- |
| Country, study year | **Responsible Institute** | **Sampling method** | **If a quota based method or stratification was used, quotas/stratification were set for:** | | | | | | **Exclusion criteria** |
|  |  |  | Age | Sex | Regions | Urban/ rural area | SEL | Other |  |
| Mexico, 2012 | IPSOS | Systematic random sampling, Quota method | Yes | Yes | Yes | No | Yes | No | None |
| Brazil, 2008 | GFK | Quota method | Yes | Yes | Yes | No | Yes | No | Working in company advertising ^a^ |
| Argentina, 2009 | TNS | Quota method | Yes | Yes | Yes | No | Yes | No | Working in company advertising ^a^; participating to survey about non-alcoholic drinks in last 6 months |
| Uruguay, 2012 | GFK | Quota method | Yes | Yes | Yes | No | Yes | No | Working in company advertising ^a^ |
| Spain, 2012 | TNS | Quota method | Yes | Yes | Yes | Yes | Yes | Educational level | Working in company advertising ^a^ |
| France, 2012 | TNS | Quota method | Yes | Yes | Yes | No | Yes | No | Working in company advertising ^a^ |
| Belgium, 2012 | CEDE | Random, Stratified Cluster sampling (schools) | Yes | Yes | Yes | No | No | Educational system | None |
| UK, 2010 | IPSOS | Quota method | Yes | Yes | Yes | No | Yes | No | Away from home for more than two nights in the sampling week ; specific diagnosed disease; following a medically-prescribed diet |
| Poland, 2014 | TNS | Quota method | Yes | Yes | Yes | Yes | Yes | Educational level | Working in company advertising ^a^; participating in market research within last 6 months |
| Turkey, 2011 | IPSOS | Systematic random sampling, Quota method | Yes | Yes | Yes | No | Yes | No | None |
| Iran, 2013 | NNFTRI | Random, stratified Cluster sampling (schools) | No | Yes | Yes | No | Yes | School grade | None |
| China, 2011 | CDC | Multi-stage random sampling | No | No | No | Yes | No | School grade | Specific diagnosed disease |
| Indonesia, 2012 | Nielsen | Systematic random sampling, Quota method | Yes | Yes | No | Yes | Yes | No | Working in company advertising ^a^ |

^a^ Working in company advertising, marketing, market research, media or manufacture, distribution and sale of different types of beverages

Abbreviations: CDC Center of Disease Control; CEDE Club Européen des Diététiciens de l’Enfance; NNFTRI National Nutrition and Food Technology Research Institute, Shahid Beheshti University of Medical Sciences, Tehran, Iran; SEL socio-economic level.

| b. Data collection | | | | | |
| --- | --- | --- | --- | --- | --- |
| Country | **Period of data collection** | **Recruited age range** | **Age recording** | **Dietary assessment method** | **Administration form** |
| Mexico, 2012 | April – May | 1-65 | Continuous | 7 day fluid record | Paper |
| Brazil, 2008 | Wave 1: March Wave 2: September | 1-55 | Continuous | 2 day fluid record | Paper |
| Argentina, 2012 | November - December | 0-65 | Continuous | 7 day fluid record ^b^ | Paper |
| Uruguay, 2012 | Wave 1: March Wave 2: December | 6-65 | Categorical | 7 day fluid record | Paper |
| Spain, 2012 | March - May | 6-70 | Continuous | 7 day fluid record | Paper |
| France, 2012 | April | 0-70 | Continuous | 7 day fluid record | Online |
| Belgium, 2012 | February - June | 8-13 | Continuous | 7 day fluid record | Paper |
| UK, 2010 | May | 3-65 | Continuous | 7 day fluid record | Paper |
| Poland, 2014 | May | 3-87 | Continuous | 7 day fluid record | Paper |
| Turkey, 2011 | January – February | 2-55 | Continuous | 7 day fluid record | Paper |
| Iran, 2013 | April – May | 8 - 17 | Continuous | 7 day fluid record ^b^ | Paper |
| China, 2011 | September – October | 8 – 17 | Continuous | 7 day fluid record | Paper |
| Indonesia, 2012 | June – July | 1- 65 | Continuous | 7 day fluid record ^b^ | Paper |

^b^ Non-alcoholic beverages only. Abbreviation: n.a. not available
