# Supplementary material for: Intake of water and beverages of children and adolescents in 13 countries
Source: Eur J Nutr. 2015 Jun 14;54(Suppl 2):69–79. doi: 10.1007/s00394-015-0955-5 (PMC4473084; doi:10.1007/s00394-015-0955-5)
Supplement: Supplementary file 2 — Supplementary material 2 (DOCX 17 kb) [file 394_2015_955_MOESM2_ESM.docx]

**Annex 2.** Classification of the fluid types

| **Classification  of fluids** | **Detailed Fluid types** |
| --- | --- |
| Water | Still water, unflavored sparkling water, tap/filtered/boiled water |
| Milk  and derivatives | Low fat and full fat milk, fermented milk, ready-to-drink milk, flavored milk, yogurt milk, *atole/champurado*, raw milk, powder milk, powder/syrup flavored milk, fruit shake with milk, cocoa compound with milk |
| Hot beverages | Coffee, coffee from coffee maker (e.g.: homemade coffee, dolce gusto, others), powder coffee, instant coffee, vending machine coffee, restaurant/franchise coffee, homemade hot/cold tea (from tea bags), Infusions (herbal), |
| Juices | Packages fruits & vegetables juices (packages juices (fruits or vegetables), packaged orangeade, packages nectars, Eskimos/smoothies), *aguas frescas*, natural fruits & vegetables juices (natural fruit/vegetable juices, restaurant lemonade/orangeade), Sugarcane juice |
| Regular soft beverages | Cola carbonated drinks, flavored carbonated drinks, flavored sparkling water, tonic, soda, flavored packaged water, flavored waters made with powder or concentrate/syrup, fruit shake with water powder, ready to drink tea, ready to drink ice tea, sports drinks (such as Aquarius, etc), vitamin/ functional drinks (fibre, vitamin and cooling drinks such as C1000 Vitamin Lemon), energy drinks, cocoa compound with water, diet sweet beverages (packaged light juices, diet/light/zero cola carbonated drinks, diet/light/zero flavored carbonated drinks) |
| Alcoholic  beverages | Beer, malt beer, beer mix drinks, wine, champagne, aperitifs and digestives straight, packages/canned alcoholic beverages, alcoholic beverages straight (not mixed like vodka, tequila, rum, ….), aperitifs and digestives with other beverages |
| Other beverages | - Beverages identified by participants as “other than listed above” - Packaged soy drinks, traditional Indonesian drinks, Jamu (Indonesia), Agua de arroz (Mexico), diet drinks as meal replacement (slim fast), Ready to drink soy based juice, Ayran (Turkey) |
