# Supplementary material for: Intake of water and beverages of children and adolescents in 13 countries
Source: Eur J Nutr. 2015 Jun 14;54(Suppl 2):69–79. doi: 10.1007/s00394-015-0955-5 (PMC4473084; doi:10.1007/s00394-015-0955-5)
Supplement: Supplementary file 3 — Supplementary material 3 (DOCX 49 kb) [file 394_2015_955_MOESM3_ESM.docx]

**Annex 3.** Percentiles of daily intake of different types of fluid intake (ml/day) stratified by country and age group

|  | Children (4-9 years) | | | | | | | | Adolescents (10-17 years) | | | | | | | |
| --- | --- | --- | --- | --- | --- | --- | --- | --- | --- | --- | --- | --- | --- | --- | --- | --- |
|  | *SEM* | *Percentiles* | | | | | | | *SEM* | *Percentiles* | | | | | | |
|  |  | *5* | *10* | *25* | *50* | *75* | *90* | *95* |  | *5* | *10* | *25* | *50* | *75* | *90* | *95* |
| Mexico |  |  |  |  |  |  |  |  |  |  |  |  |  |  |  |  |
| Water | 21 | 0 | 44 | 139 | 314 | 579 | 868 | 1305 | 30 | 0 | 36 | 152 | 364 | 575 | 1006 | 1466 |
| Milk & derivatives | 12 | 21 | 71 | 171 | 310 | 464 | 618 | 776 | 16 | 0 | 0 | 125 | 300 | 491 | 665 | 780 |
| Hot beverages | 4 | 0 | 0 | 0 | 0 | 19 | 98 | 156 | 7 | 0 | 0 | 0 | 0 | 71 | 205 | 324 |
| Juices | 11 | 0 | 0 | 0 | 86 | 214 | 398 | 547 | 12 | 0 | 0 | 0 | 97 | 237 | 444 | 638 |
| RSB | 19 | 0 | 64 | 161 | 304 | 520 | 786 | 1166 | 26 | 0 | 51 | 173 | 351 | 604 | 1013 | 1285 |
| Alcoholic beverages | 0 | 0 | 0 | 0 | 0 | 0 | 0 | 0 | 1 | 0 | 0 | 0 | 0 | 0 | 0 | 0 |
| Other beverages | 7 | 0 | 0 | 0 | 0 | 0 | 0 | 21 | 1 | 0 | 0 | 0 | 0 | 0 | 0 | 28 |
| Brazil |  |  |  |  |  |  |  |  |  |  |  |  |  |  |  |  |
| Water | 19 | 0 | 150 | 300 | 500 | 700 | 1000 | 1200 | 26 | 0 | 100 | 350 | 600 | 900 | 1324 | 1611 |
| Milk & derivatives | 15 | 50 | 150 | 290 | 403 | 600 | 800 | 1000 | 13 | 0 | 0 | 100 | 288 | 500 | 700 | 800 |
| Hot beverages | 5 | 0 | 0 | 0 | 0 | 0 | 175 | 300 | 10 | 0 | 0 | 0 | 0 | 150 | 300 | 423 |
| Juices | 19 | 50 | 100 | 200 | 400 | 675 | 950 | 1188 | 22 | 0 | 100 | 300 | 500 | 850 | 1200 | 1550 |
| RSB | 12 | 0 | 0 | 0 | 0 | 200 | 400 | 650 | 17 | 0 | 0 | 0 | 150 | 400 | 763 | 1000 |
| Alcoholic beverages | 0 | 0 | 0 | 0 | 0 | 0 | 0 | 0 | 5 | 0 | 0 | 0 | 0 | 0 | 0 | 0 |
| Other beverages | 2 | 0 | 0 | 0 | 0 | 0 | 0 | 0 | 1 | 0 | 0 | 0 | 0 | 0 | 0 | 0 |
| Uruguay |  |  |  |  |  |  |  |  |  |  |  |  |  |  |  |  |
| Water | 66 | 153 | 300 | 406 | 750 | 1100 | 1500 | 2100 | 65 | 125 | 200 | 400 | 700 | 1125 | 1650 | 2013 |
| Milk & derivatives | 28 | 200 | 300 | 429 | 500 | 744 | 875 | 949 | 31 | 125 | 200 | 300 | 475 | 650 | 900 | 1050 |
| Hot beverages | 5 | 0 | 0 | 0 | 0 | 0 | 54 | 124 | 46 | 0 | 0 | 0 | 0 | 105 | 500 | 700 |
| Juices | 60 | 0 | 0 | 25 | 200 | 556 | 1044 | 1619 | 43 | 0 | 0 | 0 | 250 | 700 | 1000 | 1100 |
| RSB | 55 | 0 | 0 | 100 | 400 | 591 | 1034 | 1290 | 58 | 0 | 0 | 300 | 573 | 900 | 1313 | 1565 |
| Alcoholic beverages | 0 | 0 | 0 | 0 | 0 | 0 | 0 | 0 | 16 | 0 | 0 | 0 | 0 | 0 | 0 | 385 |
| Other beverages | 17 | 0 | 0 | 0 | 0 | 0 | 0 | 0 | 5 | 0 | 0 | 0 | 0 | 0 | 0 | 0 |
| Argentina |  |  |  |  |  |  |  |  |  |  |  |  |  |  |  |  |
| Water | 59 | 0 | 0 | 0 | 214 | 514 | 1018 | 1529 | 32 | 0 | 0 | 0 | 177 | 453 | 761 | 896 |
| Milk & derivatives | 18 | 54 | 144 | 231 | 346 | 468 | 541 | 649 | 17 | 0 | 32 | 94 | 180 | 324 | 483 | 512 |
| Hot beverages | 32 | 0 | 0 | 0 | 34 | 140 | 373 | 688 | 45 | 0 | 0 | 0 | 183 | 473 | 1015 | 1197 |
| Juices | 40 | 0 | 0 | 11 | 203 | 426 | 779 | 1142 | 40 | 0 | 0 | 0 | 150 | 422 | 785 | 1010 |
| RSB | 67 | 0 | 50 | 157 | 514 | 801 | 1305 | 1824 | 75 | 0 | 59 | 213 | 429 | 854 | 1210 | 2319 |
| Alcoholic beverages | ND | ND | ND | ND | ND | ND | ND | ND | ND | ND | ND | ND | ND | ND | ND | ND |
| Other beverages | 1 | 0 | 0 | 0 | 0 | 0 | 0 | 0 | 3 | 0 | 0 | 0 | 0 | 0 | 0 | 60 |
| Spain |  |  |  |  |  |  |  |  |  |  |  |  |  |  |  |  |
| Water | 57 | 131 | 238 | 468 | 673 | 974 | 1611 | 1916 | 57 | 191 | 250 | 486 | 786 | 1245 | 1566 | 2065 |
| Milk & derivatives | 30 | 38 | 163 | 300 | 500 | 670 | 874 | 1054 | 23 | 0 | 0 | 231 | 326 | 503 | 702 | 930 |
| Hot beverages | 11 | 0 | 0 | 0 | 0 | 0 | 151 | 315 | 10 | 0 | 0 | 0 | 0 | 34 | 189 | 262 |
| Juices | 33 | 0 | 0 | 52 | 143 | 271 | 424 | 669 | 22 | 0 | 0 | 17 | 119 | 285 | 486 | 622 |
| RSB | 16 | 0 | 0 | 0 | 18 | 132 | 365 | 434 | 33 | 0 | 0 | 27 | 141 | 349 | 572 | 934 |
| Alcoholic beverages | 0 | 0 | 0 | 0 | 0 | 0 | 0 | 0 | 3 | 0 | 0 | 0 | 0 | 0 | 0 | 11 |
| Other beverages | ND | ND | ND | ND | ND | ND | ND | ND | ND | ND | ND | ND | ND | ND | ND | ND |
| France |  |  |  |  |  |  |  |  |  |  |  |  |  |  |  |  |
| Water | 21 | 95 | 203 | 337 | 498 | 697 | 884 | 1025 | 24 | 173 | 248 | 417 | 600 | 798 | 1045 | 1155 |
| Milk & derivatives | 12 | 0 | 0 | 115 | 250 | 334 | 450 | 533 | 13 | 0 | 0 | 118 | 225 | 311 | 436 | 581 |
| Hot beverages | 1 | 0 | 0 | 0 | 0 | 0 | 0 | 21 | 8 | 0 | 0 | 0 | 0 | 20 | 124 | 223 |
| Juices | 7 | 0 | 0 | 10 | 63 | 134 | 221 | 273 | 7 | 0 | 0 | 15 | 99 | 163 | 250 | 287 |
| RSB | 13 | 0 | 0 | 17 | 79 | 200 | 362 | 536 | 18 | 0 | 21 | 64 | 170 | 321 | 531 | 812 |
| Alcoholic beverages | 0 | 0 | 0 | 0 | 0 | 0 | 0 | 0 | 0 | 0 | 0 | 0 | 0 | 0 | 0 | 0 |
| Other beverages | ND | ND | ND | ND | ND | ND | ND | ND | ND | ND | ND | ND | ND | ND | ND | ND |
| Belgium |  |  |  |  |  |  |  |  |  |  |  |  |  |  |  |  |
| Water | 19 | 0 | 18 | 143 | 321 | 544 | 779 | 995 | 15 | 0 | 52 | 169 | 347 | 586 | 852 | 1090 |
| Milk & derivatives | 9 | 0 | 0 | 29 | 114 | 214 | 345 | 448 | 5 | 0 | 0 | 29 | 114 | 217 | 303 | 371 |
| Hot beverages | ND | ND | ND | ND | ND | ND | ND | ND | ND | ND | ND | ND | ND | ND | ND | ND |
| Juices | 8 | 0 | 0 | 31 | 93 | 189 | 310 | 435 | 7 | 0 | 0 | 21 | 107 | 226 | 338 | 449 |
| RSB | 11 | 0 | 0 | 29 | 107 | 239 | 409 | 591 | 9 | 0 | 0 | 64 | 171 | 315 | 479 | 645 |
| Alcoholic beverages | 0 | 0 | 0 | 0 | 0 | 0 | 0 | 0 | 0 | 0 | 0 | 0 | 0 | 0 | 0 | 0 |
| Other beverages | 2 | 0 | 0 | 0 | 0 | 29 | 76 | 105 | 2 | 0 | 0 | 0 | 0 | 18 | 86 | 130 |
| UK |  |  |  |  |  |  |  |  |  |  |  |  |  |  |  |  |
| Water | 41 | 0 | 28 | 159 | 375 | 639 | 928 | 1431 | 31 | 14 | 50 | 148 | 366 | 687 | 1136 | 1406 |
| Milk & derivatives | 23 | 0 | 0 | 0 | 208 | 398 | 627 | 816 | 14 | 0 | 0 | 28 | 103 | 260 | 490 | 656 |
| Hot beverages | 10 | 0 | 0 | 0 | 0 | 37 | 195 | 332 | 18 | 0 | 0 | 0 | 23 | 189 | 581 | 781 |
| Juices | 22 | 0 | 0 | 30 | 214 | 382 | 538 | 700 | 22 | 0 | 0 | 26 | 151 | 334 | 672 | 859 |
| RSB | 41 | 0 | 0 | 117 | 438 | 768 | 1234 | 1380 | 35 | 0 | 77 | 223 | 516 | 778 | 1171 | 1689 |
| Alcoholic beverages | 0 | 0 | 0 | 0 | 0 | 0 | 0 | 0 | 5 | 0 | 0 | 0 | 0 | 0 | 0 | 0 |
| Other beverages | 0 | 0 | 0 | 0 | 0 | 0 | 0 | 0 | 2 | 0 | 0 | 0 | 0 | 0 | 0 | 39 |
| Poland |  |  |  |  |  |  |  |  |  |  |  |  |  |  |  |  |
| Water | 26 | 0 | 0 | 16 | 199 | 357 | 596 | 943 | 24 | 0 | 0 | 71 | 264 | 491 | 713 | 950 |
| Milk & derivatives | 11 | 0 | 0 | 29 | 143 | 245 | 349 | 396 | 10 | 0 | 0 | 0 | 71 | 207 | 294 | 402 |
| Hot beverages | 21 | 21 | 143 | 327 | 457 | 573 | 761 | 907 | 22 | 100 | 179 | 286 | 454 | 607 | 796 | 1038 |
| Juices | 14 | 0 | 0 | 55 | 163 | 286 | 470 | 550 | 14 | 0 | 0 | 36 | 152 | 274 | 445 | 559 |
| RSB | 20 | 0 | 0 | 125 | 253 | 395 | 611 | 813 | 23 | 0 | 0 | 95 | 263 | 484 | 693 | 875 |
| Alcoholic beverages | 0 | 0 | 0 | 0 | 0 | 0 | 0 | 0 | 2 | 0 | 0 | 0 | 0 | 0 | 0 | 30 |
| Other beverages | 7 | 0 | 0 | 0 | 0 | 0 | 36 | 71 | 8 | 0 | 0 | 0 | 0 | 0 | 36 | 77 |
| Turkey |  |  |  |  |  |  |  |  |  |  |  |  |  |  |  |  |
| Water | 34 | 357 | 404 | 555 | 771 | 1012 | 1343 | 1796 | 37 | 291 | 409 | 611 | 835 | 1172 | 1647 | 2165 |
| Milk & derivatives | 24 | 0 | 0 | 43 | 171 | 450 | 690 | 872 | 17 | 0 | 0 | 0 | 90 | 286 | 532 | 758 |
| Hot beverages | 14 | 0 | 3 | 65 | 182 | 284 | 481 | 537 | 15 | 0 | 23 | 110 | 204 | 363 | 540 | 704 |
| Juices | 11 | 0 | 0 | 13 | 73 | 194 | 335 | 445 | 13 | 0 | 0 | 0 | 63 | 163 | 349 | 497 |
| RSB | 17 | 0 | 0 | 0 | 46 | 138 | 329 | 571 | 14 | 0 | 0 | 21 | 115 | 248 | 422 | 537 |
| Alcoholic beverages | 0 | 0 | 0 | 0 | 0 | 0 | 0 | 0 | 1 | 0 | 0 | 0 | 0 | 0 | 0 | 0 |
| Other beverages | 19 | 0 | 0 | 3 | 107 | 214 | 470 | 545 | 18 | 0 | 0 | 33 | 129 | 271 | 498 | 827 |
| Iran |  |  |  |  |  |  |  |  |  |  |  |  |  |  |  |  |
| Water | 24 | 208 | 276 | 416 | 598 | 868 | 1122 | 1269 | 16 | 154 | 215 | 406 | 599 | 853 | 1229 | 1414 |
| Milk & derivatives | 13 | 30 | 74 | 169 | 247 | 378 | 509 | 619 | 8 | 0 | 32 | 92 | 184 | 333 | 470 | 585 |
| Hot beverages | 7 | 0 | 6 | 55 | 101 | 167 | 251 | 283 | 6 | 16 | 45 | 107 | 185 | 279 | 404 | 488 |
| Juices | 6 | 0 | 0 | 8 | 57 | 113 | 197 | 248 | 3 | 0 | 0 | 0 | 30 | 84 | 147 | 205 |
| RSB | 7 | 0 | 0 | 33 | 81 | 165 | 233 | 277 | 6 | 0 | 0 | 46 | 111 | 210 | 338 | 407 |
| Alcoholic beverages | ND | ND | ND | ND | ND | ND | ND | ND | ND | ND | ND | ND | ND | ND | ND | ND |
| Other beverages | 1 | 0 | 0 | 0 | 0 | 0 | 16 | 26 | 1 | 0 | 0 | 0 | 0 | 0 | 30 | 54 |
| China |  |  |  |  |  |  |  |  |  |  |  |  |  |  |  |  |
| Water | 11 | 207 | 281 | 400 | 570 | 821 | 1114 | 1366 | 7 | 218 | 294 | 443 | 671 | 1014 | 1435 | 1756 |
| Milk & derivatives | 5 | 0 | 0 | 57 | 159 | 264 | 398 | 486 | 2 | 0 | 0 | 43 | 157 | 257 | 384 | 479 |
| Hot beverages | 2 | 0 | 0 | 0 | 0 | 0 | 29 | 86 | 2 | 0 | 0 | 0 | 0 | 0 | 82 | 167 |
| Juices | 3 | 0 | 0 | 0 | 0 | 57 | 129 | 212 | 2 | 0 | 0 | 0 | 0 | 89 | 214 | 333 |
| RSB | 3 | 0 | 0 | 0 | 0 | 71 | 162 | 243 | 2 | 0 | 0 | 0 | 0 | 86 | 214 | 307 |
| Alcoholic beverages | ND | ND | ND | ND | ND | ND | ND | ND | ND | ND | ND | ND | ND | ND | ND | ND |
| Other beverages | 1 | 0 | 0 | 0 | 0 | 13 | 57 | 99 | 1 | 0 | 0 | 0 | 0 | 11 | 69 | 115 |
| Indonesia |  |  |  |  |  |  |  |  |  |  |  |  |  |  |  |  |
| Water | 37 | 295 | 567 | 871 | 1243 | 1885 | 2458 | 2825 | 34 | 428 | 601 | 951 | 1474 | 2137 | 2791 | 3120 |
| Milk & derivatives | 13 | 0 | 0 | 0 | 103 | 287 | 536 | 678 | 6 | 0 | 0 | 0 | 0 | 79 | 228 | 319 |
| Hot beverages | 8 | 0 | 0 | 0 | 0 | 105 | 262 | 328 | 8 | 0 | 0 | 0 | 23 | 174 | 322 | 448 |
| Juices | 4 | 0 | 0 | 0 | 0 | 0 | 59 | 141 | 3 | 0 | 0 | 0 | 0 | 0 | 57 | 138 |
| RSB | 17 | 0 | 0 | 0 | 69 | 213 | 447 | 647 | 15 | 0 | 0 | 0 | 80 | 219 | 540 | 911 |
| Alcoholic beverages | ND | ND | ND | ND | ND | ND | ND | ND | ND | ND | ND | ND | ND | ND | ND | ND |
| Other beverages | 2 | 0 | 0 | 0 | 0 | 0 | 38 | 105 | 3 | 0 | 0 | 0 | 0 | 0 | 30 | 110 |
| TOTAL SAMPLE |  |  |  |  |  |  |  |  |  |  |  |  |  |  |  |  |
| Water | 9 | 34 | 129 | 307 | 542 | 857 | 1309 | 1711 | 6 | 100 | 200 | 393 | 774 | 1005 | 1510 | 1904 |
| Milk & derivatives | 4 | 0 | 0 | 79 | 214 | 379 | 571 | 700 | 2 | 0 | 0 | 36 | 191 | 279 | 436 | 554 |
| Hot beverages | 3 | 0 | 0 | 0 | 0 | 36 | 214 | 374 | 2 | 0 | 0 | 0 | 76 | 71 | 255 | 400 |
| Juices | 4 | 0 | 0 | 0 | 50 | 193 | 400 | 600 | 2 | 0 | 0 | 0 | 121 | 146 | 346 | 544 |
| RSB | 5 | 0 | 0 | 0 | 71 | 239 | 500 | 741 | 3 | 0 | 0 | 0 | 161 | 207 | 436 | 675 |
| Alcoholic beverages | 0 | 0 | 0 | 0 | 0 | 0 | 0 | 0 | 1 | 0 | 0 | 0 | 5 | 0 | 0 | 0 |
| Other beverages | 1 | 0 | 0 | 0 | 0 | 0 | 51 | 107 | 1 | 0 | 0 | 0 | 21 | 0 | 64 | 125 |

Abbreviations: ND no data, RSB regular soft beverages, SEM standard error of the mean
